# The quality of maternity care services as experienced by women in the Netherlands

**DOI:** 10.1186/1471-2393-9-18

**Published:** 2009-05-09

**Authors:** Therese A Wiegers

**Affiliations:** 1 NIVEL (Netherlands institute for health services research), P.O. Box 1568, 3500 BN Utrecht, The Netherlands

## Abstract

**Background:**

Maternity care is all care in relation to pregnancy, childbirth and the postpartum period. In the Netherlands maternity care is provided by midwives and general practitioners (GPs) in primary care and midwives and gynecologists in secondary care. To be able to interpret women's experience with the quality of maternity care, it is necessary to take into account their 'care path', that is: their route through the care system.

In the Netherlands a new tool is being developed to evaluate the quality of care from the perspective of clients. The tool is called: 'Consumer Quality Index' or CQI and is, within a standardized and systematic framework, tailored to specific health care issues.

Within the framework of developing a CQI Maternity Care, data were gathered about the care women in the Netherlands received during pregnancy, childbirth, and the postpartum period. In this paper the quality of maternity care in the Netherlands is presented, as experienced by women at different stages of their care path.

**Methods:**

A sample of 1,248 pregnant clients of four insurance companies, with their due date in early April 2007, received a postal survey in the third trimester of pregnancy (response 793). Responders to the first questionnaire received a second questionnaire twelve weeks later, on average four weeks after delivery (response 632). Based on care provider and place of birth the 'care path' of the women is described. With factor analysis and reliability analysis five composite measures indicating the quality of treatment by the care provider at different stages of the care path have been constructed. Overall ratings relate to eight different aspects of care, varying from antenatal care by a midwife or GP to care related to neonatal screening.

**Results:**

41.5 percent of respondents remained in primary care throughout pregnancy, labor, birth and the postpartum period, receiving care from a midwife or general practitioner, 31.3% of respondents gave birth at home. The majority of women (58.5%) experienced referral from one care provider to another, i.e. from primary to secondary care or reverse, at least once. All but two percent of women had one or more ultrasound scans during pregnancy. The composite measures for the quality of treatment in different settings and by different care providers showed that women, regardless of parity, were very positive about the quality of the maternity care they received. Quality-of-treatment scores were high: on average 3.75 on a scale ranging from 1 to 4. Overall ratings on a 0 – 10 scale for quality of care during the antenatal period and during labor, birth and the postpartum period were high as well, on average 8.36.

**Conclusion:**

The care path of women in maternity care was seldom straight forward. The majority of pregnant women switched from primary to secondary care and back at least once, during pregnancy or during labor and birth or both.

The results of the quality measures indicate that the quality of care as experienced by women is high throughout the care system. But with regard to the care during labor and birth the quality of care scores are higher when women know their care provider, when they give birth at home, when they give birth in primary care and when they are assisted by their own midwife.

## Background

Maternity care in the Netherlands is different from maternity care in most other countries, not only because of the relatively high percentage of home births, but also because of the autonomy of the midwife, as medical professional, and the structure of the Dutch health care system with a clear boundary between primary and secondary care [1-3]. In the Netherlands people with health complaints or concerns are expected to see a general practitioner (GP), who will refer them to a medical specialist, if needed. The GP, as primary care provider, serves as gatekeeper for secondary, specialist care. Secondary care, provided by medical specialists in hospitals or clinics, is only accessible after referral from a primary care provider.

In maternity care the primary care provider and gatekeeper is a midwife, although some GPs still provide (part of the) care during pregnancy and childbirth. Most primary care midwives work in group practices and are jointly responsible for their clients (for more background on the work of midwives in the Netherlands: see [2]). A healthy woman with an uncomplicated pregnancy has no need to see another care provider than her midwife, and she can freely choose where to give birth, at home or in a hospital or birth centre. There she will be attended by her own midwife (or her colleague) or GP, without supervision of a gynecologist. However, whenever and as long as there is an increased risk of complications, requiring specialist care, the woman will have to consult a gynecologist in secondary care. This means that at any moment during pregnancy or childbirth a woman can be referred from primary to secondary care and back. Postpartum care is provided by midwives (or occasionally GPs) and maternity care assistants (MCAs) unless the woman and/or her baby is hospitalized and a gynecologist and/or neonatologist is responsible. This means that the 'care path' in maternity care can be straight forward when pregnancy and birth are uncomplicated, but can become complicated otherwise.

In maternity care the client-centered approach has led to increased activity to measure women's satisfaction, preferences and experiences, in the Netherlands [4,5] as well as elsewhere, as is shown by reports as: Listening to Mothers I and II [6,7], Giving Birth in Canada [8], Recorded delivery [9] and Women's experiences of maternity care in the NHS [10]. These reports not only show how women evaluate the care they received, but they also underscore the complexity of maternity care and the many different routes, or 'care paths' women can take through the health care system in the different countries. The studies aim to understand and improve the quality of maternity services, by obtaining not only information on outcome indicators, such as mortality, morbidity, and satisfaction, but also information about women's views of and experiences with structure and process indicators of care. As Redshaw writes: 'allowing women to express their views on different phases of care, on the care provided by different health professionals and in different settings (....), provides a richer and more realistic picture of the care they received' [11].

Studies about client satisfaction are abundant, but their relation to studies about quality of maternity care shows contradictory results [12] and the connection between satisfaction and quality of care is often confusing).) [13-15]. One of the reasons is that client satisfaction is only indirectly related to the quality of the health care system, because it is strongly colored by expectations and prior experiences. It is shown that users tend to value what is available and known to them more than what is new and unexpected [16]. Because satisfaction with care is generally high, regardless of the quality of the care provided, a different approach is developed. The input of clients in the quality of care discussion has been redefined [17] and has shifted from client satisfaction to client experience, that is: to the assessment of health care quality from the patient's perspective. Since the mid-1990s the CAHPS^® ^Consortium in the USA (CAHPS^® ^stands for: Consumer Assessment of Healthcare Providers and Systems) has been developing instruments that measure clients' actual experience with health care services, as well as general ratings of these services [18].

Also in the 1990s NIVEL (Netherlands institute for health services research) developed a series of QUOTE-questionnaires (QUOTE stands for: QUality Of care Through the patient's Eyes), assessing opinions, ideas, wishes and experiences of clients with regard to quality of care, based on what is regarded to be important by clients, to be taken into account in organizations' policies to improve the quality of care [19]. Recently a new measurement instrument: the Consumer Quality Index or CQI, based on both the CAHPS^® ^and the QUOTE instruments, is being developed in the Netherlands [20]. The CQI aims to measure the actual experience of clients with specific structure and process aspects of health care, such as treatment, accessibility and information, as well as the importance clients attach to each aspect. The combination of importance with experience can give health care providers an indication of aspects that may need improvement. For each issue or theme the developing process includes: focus group discussions with clients and discussions with other stakeholders (care providers, insurers), drafting the questionnaire, data collection, testing psychometric properties (factor analysis, reliability analysis), adjusting the questionnaire, and finally testing whether the tool can be used to distinguish between providers on the basis of client experience of the quality of care.

Within the framework of developing a CQI Maternity Care, data were gathered about the care women in the Netherlands received during pregnancy, childbirth, and the postpartum period. In this paper the quality of maternity care in the Netherlands is presented, as experienced by women at different stages of their care path.

## Methods

In order to develop a CQI questionnaire to measure the quality of maternity care [21] a survey was conducted early in 2007, funded by Miletus, a consortium of (then) four insurance companies. The content of the survey was based on focus group discussions with maternity care users (antenatal and postnatal) about their expectations and experiences of the quality of maternity care, and information from care providers and insurance companies. The survey consisted of postal questionnaires, one to be completed in the third trimester of pregnancy, the other to be completed a few weeks after the baby was born. Identifiers made it possible to link both surveys. No ethical approval is required in the Netherlands for survey research. To protect women's confidentiality introductory letters and questionnaires were sent through a third party: a mail house. Women who wished to participate returned the completed questionnaire in a prepaid envelope to the mail house.

A sample of 1.248 pregnant women, drawn from the client register of the participating insurance companies, based on their application for maternity care assistance (MCA) and an expected date of birth in early April 2007, received the first (antenatal) questionnaire in February (two months before their due date). After two reminders the response was 64% (793/1.248). The second (postnatal) questionnaire was sent 3 months after the first, only to those women who had responded to the first questionnaire. After three reminders the response was 80% (632/739), leading to a total net response of 51% (632/1.248). The number for specific variables, presented in the tables, may be slightly less due to missing data.

The response group is representative for women giving birth in the Netherlands in 2006 with regard to age (an average of 31 years) and parity (45% first-time mothers) [22]. No other data are available to compare representativeness. But, as the sample was drawn from the register of four of the largest health insurance companies in the country, and as every resident in the Netherlands is by law obliged to be insured, we can assume the sample to be reasonably representative for women giving birth in the Netherlands.

The questionnaires combined ***informative questions ***(what happened?) concerning prenatal care, referral to secondary care, ultrasound scans and antenatal screening, birth preparation, labor and birth, hospital stay, postpartum period, and neonatal screening, with ***evaluative questions ***(how often did you experience...?) with answers ranging from 1 = never to 4 = always, and ***general ratings***, ranging from 0 = worst possible care to 10 = best possible care.

The informative questions provided the information about the care path of women in maternity care. In the Netherlands most pregnant women are offered a dating scan in early pregnancy. Since January 2006 all pregnant women are entitled to a 20-week or 'anomaly' scan but screening for Down's syndrome may only be offered to women of 36 years or older or on the basis of a medical indication. Because there are no data available on the actual use of ultrasound in pregnancy, a question was added to the questionnaire. The evaluative questions and the general ratings provided information about the quality of care from the perspective of women. The evaluative questions were based on quality indicators about treatment by a particular caregiver at a particular time (feeling in safe hands, having things explained in an understandable way, being treated with respect, being listened to carefully, being taken seriously, being given enough time, being given enough opportunity to ask questions). Five composite measures were constructed which were found to be reliable: (a) quality of treatment by the midwife or GP during pregnancy (7 items, Cronbach's alpha 0.87), (b) quality of treatment by the gynecologist during pregnancy (7 items, Cronbach's alpha 0.92), (c) quality of treatment during labor and birth (7 items, Cronbach's alpha 0.90), (d) quality of treatment by the midwife or GP during postnatal care (7 items, Cronbach's alpha 0.89), (e) quality of treatment by the maternity care assistant (MCA) during the postpartum period (11 items, Cronbach's alpha 0.95; additional items are: including your partner in the care, feeling your baby is in safe hands, spending enough time with the baby, spending enough time with the rest of the family).

General ratings were asked for eight different aspects of care: antenatal care by midwife or GP, antenatal care by gynecologist, care in relation to ultrasound scans, care during labor and birth, care during hospital stay, postpartum care by midwife or GP, postpartum care by MCA, care in relation to neonatal screening. The data were analyzed using SPSS 14.0 for Windows, p-values were calculated using the Pearson Chi-Square or Fisher Exact test.

## Results

### Care path

Of the 793 women who returned the antenatal questionnaire most saw a midwife for antenatal care (see table 1). Nine percent of the respondents went directly to a gynecologist. Almost half of all pregnant women, 48 percent, saw a gynecologist at least once during their pregnancy, 12 percent for a single consultation (not in the table), 36 percent for two or more regular check-ups. About one in four women (26%) have been referred to secondary care during the course of their pregnancy.

**Table 1:** Care providers in maternity care

**All pregnant women (n = 793)**	MW*	GP*	GYN*
Which care provider did you go to first?	85.6%	5.3%	9.1%
Which care provider did you see more than once during pregnancy?**	87.5%	3.0%	36.0%

**All postpartum women (n = 632)**	MW*	GP*	GYN*

Which care provider did you see during pregnancy?**	87.5%	2.8%	35.0%
Who was your care provider at the onset of labor?	68.6%	1.6%	29.8%
Which care provider was responsible at the time of birth?	48.0%	0.8%	51.2%
Which care provider was most involved during labor/birth?	71.0%	1.1%	27.9%
Who was your care provider during the postpartum period?	90.9%	3.0%	6.1%

*MW = midwife; GP = general practitioner; GYN = gynecologist

** more than 1 response if women were referred from primary to secondary care during pregnancy

**Figure 1:**
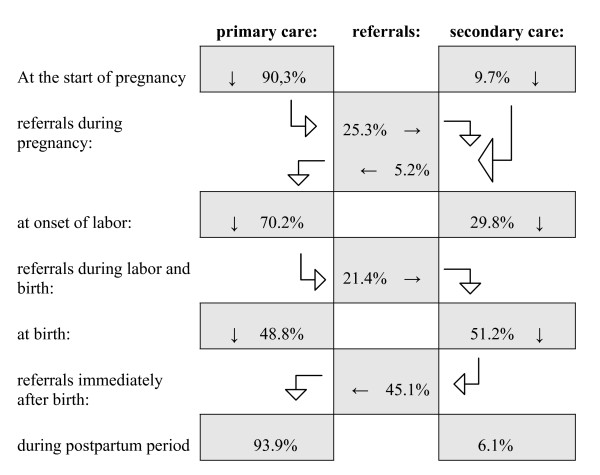
**Care path through maternity care in the Netherlands, percentage of women**.

The number of respondents who also filled out the postnatal questionnaire was 20 percent lower, but the distribution regarding the care provider during pregnancy remained similar. At the onset of labor 30 percent of respondents were already cared for in secondary care. During labor, birth, or shortly after, another 21 percent of respondents were referred from primary to secondary care. Thus, a total of 51 percent of births occurred in secondary care, supervised by gynecologists. However, in 71 percent of cases the caregiver most involved with the birthing woman was a midwife. This might have been the primary care midwife, accompanying the woman even after referral to a gynecologist, or this might have been a secondary care midwife, working under supervision of a gynecologist. Almost all women who gave birth in secondary care were referred back to primary care for the postpartum period. Figure 1 shows the care path of the women in this study. Of the women filling out the prenatal questionnaire only 2 percent did not have any ultrasound scans (see table 2). Almost one in four had one or more scans for non-medical reasons (a so-called 'fun scan', not covered by the insurance), nulliparous women significantly more often than parous women.

**Table 2:** Experience with ultrasound scans and prenatal screening

**All pregnant women (n = 776#)** (more than 1 response possible)	all women (n) %	nulliparae (n) %	Parae (n) %
no ultrasound scan	(13) 1.7%	(6) 1.7%	(7) 1.6%
anomaly scan	(572) 73.7%	(260) 74.7%	(312) 72.9%
dating scan/fetal position/threatening miscarriage	(456) 58.8%	(205) 58.9%	(251) 58.6%
Down-syndrome screening	(203) 26.2%	(97) 27.9%	(106) 24.8%
'fun'-scan**	(182) 23.5%	(99) 28.4%	(83) 19.4%
routine scan at every check-up	(111) 14.3%	(45) 12.9%	(66) 15.4%
specialized ultrasound examination	(30) 3.9%	(10) 2.9%	(20) 4.7%

# no data about parity of 17 women (see table 3)

**p < 0.01

More than one third of respondents in both parity groups planned to give birth at home (see table 3). One in four respondents had a hospital birth with a gynaecologist planned because of medical reasons. There is a significant difference between women giving birth to their first child (nulliparae) and women who have given birth before (parae) with the first group more often planning a hospital birth with their own midwife and less often being forced to a birth in secondary care because of medical reasons. The differences in actual place of birth are larger than in planned place of birth. First-time mothers more often gave birth in secondary care (61%) than women giving birth to their second or subsequent child (40%) and much less often at home (16% versus 42%).

**Table 3:** Planned and actual place of birth in relation to parity

	primary care			secondary care	
			
	at home	birth centre	hospital with own midwife	hospital with gynecologist	don't know yet/no answer/elsewhere
All pregnant women (n = 793)**					
Where are you planning to give birth?	38.7%	1.2%	26.5%	24.0%	9.7%
*nulliparae (n = 348)*	*37.6%*	*2.0%*	*31.6%*	*18.1%*	*10.6%*
*parae (n = 428)*	*39.5%*	*0.5%*	*22.4%*	*28.7%*	*8.9%*
All postpartum women (n = 632)***					
Where did you give birth?	30.6%	0.6%	15.0%	49.6%	4.2%
*primiparae (n = 280)*	*16.4%*	*0.7%*	*15.7%*	*61.1%*	*6.1%*
*multiparae (n = 341)*	*42.2%*	*0.6%*	*14.4%*	*40.2%*	*2.6%*

** Chi Square = 19.88 p < 0.01 *** Chi Square 51.54 p < 0.001

A small majority of women (54%) knew the caregiver attending them during the birth (see table 4), those giving birth at home much more often (85%) than those giving birth in a hospital or birth centre (41%) (not in table). Almost half of all births (46%) were spontaneous without intervention (episiotomies and stitches not included), fifteen percent were assisted deliveries, almost fifteen percent required induction or augmentation of labor and in twenty one percent the membranes were ruptured artificially (AROM). First-time mothers experienced more interventions, especially more assisted deliveries compared to women giving birth to their second or subsequent baby (p < 0.01). Of the 27.1% of women that used some form of pain medication, 16% received an epidural.

**Table 4:** Labor and birth assistance, interventions and outcome in relation to parity

**All postpartum women (n = 621#)**	all women yes (n) %	primiparae yes (n) %	multiparae yes (n) %
known care provider ***	(339) 54.6%	(125) 44.6%	(214) 62.6%
spontaneous birth***	(255) 41.1%	(87) 31.1%	(168) 49.3%
caesarean section	(57) 9.2%	(29) 10.4%	(28) 8.2%
vacuum/forceps**	(28) 4.5%	(20) 7.1%	(8) 2.3%
induction of labor	(52) 8.4%	(20) 7.1%	(32) 9.4%
augmentation of labor*	(29) 4.7%	(19) 6.8%	(10) 2.9%
AROM	(128) 20.6%	(55) 19.6%	(73) 21.4%
referral during labor/birth ***	(124) 20.0%	(80) 28.6%	(44) 13,9%
pain medication ***	(168) 27.1%	(115) 41.1%	(53) 15.5%

# no data about parity of 11 women (see table 3)

*p < 0.05 **p < 0.01 *** p < 0.001

### Quality of treatment and general ratings of quality of care

**Figure 2:**
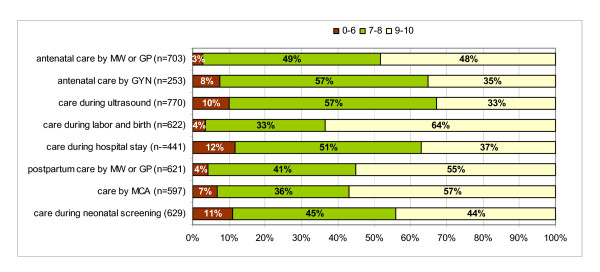
**General ratings of quality of care (three-point-scale)**.

The results on the composite measures for the quality of treatment by care providers showed that the women, regardless of parity, were very positive about the way they were treated (see table 5). The general ratings of quality of care during the antenatal period, during labor and birth, and during the postnatal period, reduced to a three-point scale (≤ 6; 7–8; 9–10) are presented in figure 2. Average ratings ranged from 8.02 (SD 1.31) for quality of care in relation to ultrasound scans, to 8.82 (SD 1.25) for quality of care during labor and birth. First-time mothers (to-be) gave lower ratings on two of the eight items: the quality of antenatal care by a midwife or GP and the quality of postnatal care by a midwife or GP. As with the composite measure for quality of treatment during labor and birth, the general rating for quality of care during labor and birth was significantly higher for women who knew their caregiver, who were assisted by their own midwife, who gave birth at home, or who gave birth in primary care (see table 6).

**Table 5:** Mean scores (SD) for quality of treatment (range 1 – 4)

**All pregnant women (n = 793)**	nulliparae (n = 348)	parae (n = 428)
Treatment by midwife or GP during pregnancy (n = 676)	3.78 (0.34)	3.80 (0.35)
Treatment by gynecologist during pregnancy (n = 236)	3.62 (0.45)	3.72 (0.40)

**All postpartum women (n = 632)**	primiparae (n = 280)	multiparae (n = 341)

Treatment during labor and birth (n = 596)	3.71 (0.49)	3.78 (0.38)
Treatment by midwife or GP during postpartum period (n = 582)	3.79 (0.38)	3.83 (0.34)
Treatment by MCA (n = 547)	3.78 (0.35)	3.73 (0.38)

**Table 6:** Scores for quality of treatment during labor and birth and general ratings of quality of care during labor and birth in relation to specific situations

	**yes**	**no**
	(n) score	(n) score
known care provider ***	(330) 3.84	(271) 3.64
giving birth at home ***	(189) 3.92	(401) 3.67
giving birth in primary care ***	(278) 3.87	(302) 3.64
assisted by own midwife ***	(292) 3.88	(255) 3.61

	**yes**	**no**
	(n) rating	(n) rating

known care provider ***	(339) 9.13	(276) 8.45
giving birth at home ***	(192) 9.33	(413) 8.58
giving birth in primary care ***	(286) 9.22	(309) 8.48
assisted by own midwife ***	(301) 9.16	(259) 8.52

score range 1 – 4 *(Chi Square test)*; general rating range 0 – 10 *(t-test);*

*** p < 0.001

## Discussion

The aim of the study was to assess women's experiences with the quality of maternity care services. To do so more insight was needed in the different care paths women can follow. This study has shown that the care path of women in maternity care in the Netherlands is seldom straight forward. Many women switched from primary to secondary care and back at least once, during pregnancy or during labor and birth or both. The majority of women started antenatal care and concluded postnatal care with a midwife in primary care, but just over half of them gave birth in a hospital, supervised by a gynecologist, and often assisted by a (secondary care) midwife. This is different from the care path of women in maternity care in the USA or Canada, where a substantial majority of women receive antenatal and natal care from a physician [7,8]. The Dutch pattern of antenatal care more resembles that in England, where approximately half of pregnant women receive antenatal care from a midwife only and where hospital doctors are involved in the care of just over a third of women [9]. The crucial difference is, however, that in the Netherlands the involvement of hospital doctors includes referral, that is: transfer of responsibility from one care provider (the midwife) to another (the gynecologist), while in England this usually means shared care. The finding that almost all women in this study had one or more ultrasound scans is not different from that in the USA and England, but in England more women had a dating scan (86%) or an anomaly scan (97%) [9]. A striking difference between the Netherlands and the USA and England is found in the number of interventions during labor and birth. For instance: in the Netherlands almost 10% of women experienced medical induction of labor, in the USA 34%, and in England 31%. Furthermore: in the Netherlands 27.5% of women received some form of pain medication, in the USA 86% and in England 93% [7,9].

## Conclusion

The composite measures for quality of treatment and the general ratings of quality of care in different settings and by different care providers showed that women, regardless of parity, were very positive about the quality of the maternity care they received. This indicates that the quality of care as experienced by women is high throughout the care system. But with regard to the care during labor and birth the quality of care scores are higher when women know their care provider, when they give birth at home, when they give birth in primary care and when they are assisted by their own midwife.

### Strengths and limitations

In order to develop a CQI instrument to evaluate maternity care as a whole, questionnaires were designed to cover many aspects of maternity care, in order to provide every respondent with an opportunity to relate her experiences. The result, however, was a wide variety of experiences, indicating a number of different care paths. The results therefore show aspects of quality of care as experienced by women in different care settings. It was not possible to give a detailed picture of the representativeness of the sample, due to the lack of comparable studies. There is indication that women with uncomplicated births were slightly overrepresented in the sample: 31.3% gave birth at home, with a estimated national home birth rate of 29% [22].

## Competing interests

The author declares there are no competing interests.

The study is funded by Miletus, a consortium of insurance companies, at the time of the study consisting of Agis, VGZ, Menzis, en Ohra/Delta Lloyd.

## Pre-publication history

The pre-publication history for this paper can be accessed here:


